# Literary Periodicals

**Published:** 1872-04

**Authors:** 


### LITERARY PERIODICALS.

SCRIBNER'S MONTHLY.—The April number of this first-class Magazine comes to us filled with its usual variety of literature. There is no better Literary Monthly than Scribner's. The illustrations of the Yosemite Valley and the Yellow-stone, in the January and February numbers are alone worth the subscription price. In the May number its readers are promised a series of illustrations, being designs by a group of the finest artists in the country, to be called "Travelling by Telegraph," besides three serials of unusual interest by some of the best writers of fiction. Address Scribner & Co., 654, Broadway, New York. Price, $4 00.

DEMOREST'S MONTHLY AND YOUNG AMERICA—Are indispensible to every well regulated household—so say the ladies and children. The first is devoted to fashion and "how to dress well;" the second, is the freshest and nicest child's periodical in the country—one that will instruct, amuse and delight the boys and girls.

SCIENTIFIC AMERICAN.—This highly useful and practical Weekly, maintains its past reputation as the best journal of its character in the world. It is always brim-full of valuable articles on Science and Art, besides being employed, constantly, in bringing before its readers, the very latest mechanical inventions and improvements. It always contains articles of value to Scientific men, and gives our people the only means of becoming well informed concerning the mechanical improvements of the age. Address Munn & Co., 37 Park Row, New York.

THE HEARTH AND HOME—Is publishing a splendid serial story, entitled "The End of the World," by the author of that life-like picture of Western life, "The Hoosier Schoolmaster." This story, with its other attractions, make this Weekly Illustrated paper the best in the country.

